# Genome Sequence of the Dichloromethane-Degrading Bacterium *Hyphomicrobium* sp. Strain GJ21

**DOI:** 10.1128/genomeA.00622-17

**Published:** 2017-07-27

**Authors:** Françoise Bringel, Christiaan P. Postema, Sophie Mangenot, Sabrina Bibi-Triki, Pauline Chaignaud, Muhammad Farhan Ul Haque, Christelle Gruffaz, Louis Hermon, Yousra Louhichi, Bruno Maucourt, Emilie E. L. Muller, Thierry Nadalig, Aurélie Lajus, Zoé Rouy, Claudine Médigue, Valérie Barbe, Dick B. Janssen, Stéphane Vuilleumier

**Affiliations:** a Université de Strasbourg, CNRS, GMGM UMR 7156, Department of Microbiology, Genomics and the Environment, Strasbourg, France; b Biochemical Laboratory, Groningen Biomolecular Sciences and Biotechnology Institute, University of Groningen, Groningen, The Netherlands; c Commissariat à l’Énergie Atomique et aux Énergies Alternatives, Institut de Biologie François-Jacob, Laboratoire de Biologie Moléculaire pour l’Etude des Génomes, Évry, France; d Commissariat à l’Énergie Atomique et aux Énergies Alternatives, Institut de Biologie François-Jacob, Laboratoire d’Analyse Bioinformatique en Génomique et Métabolisme, Évry, France; e Centre National de la Recherche Scientifique, UMR 8030, Évry, France; f UEVE, Université d’Évry, Évry, France

## Abstract

The genome sequence of *Hyphomicrobium* sp. strain GJ21, isolated in the Netherlands from samples of environments contaminated with halogenated pollutants and capable of using dichloromethane as its sole carbon and energy source, was determined.

## GENOME ANNOUNCEMENT

Dichloromethane (DCM) is one of the most used industrial halogenated solvents worldwide, and its toxicity and substantial release into the environment are of serious concern (1, 2). Various methylotrophic bacterial strains capable of growing with DCM as the sole source of carbon and energy have been isolated over the years (3). All such strains for which the dehalogenase has been characterized, including strain GJ21 (4), contain the *dcmA* gene for DCM dehalogenase, an enzyme of the glutathione *S*-transferase family (5, 6). Other DCM-degrading systems, particularly from anaerobic environments, are currently being investigated in detail (7–10), including at the genome level. Whereas natural sources of DCM exist, such as production by microalgae (11), most DCM in the environment is of industrial origin, raising questions about the origin, evolution, and distribution of DCM dehalogenase genes in microorganisms.

*Hyphomicrobium* sp. strain GJ21, isolated after prolonged adaptation from a mixture of activated sludge from a wastewater treatment plant and soil samples from polluted sites (12), has been extensively used as a model for bioremediation of DCM in bioreactor studies (e.g., see reference 13 and references therein). The genome of *Hyphomicrobium* sp. strain GJ21 was sequenced using Illumina technology. A mate-paired library (7-kb insert size) and a paired-end library (340-bp insert size) were produced and sequenced using HiSeq2000 (2 × 100 nucleotides), yielding ~4 Gb and ~1.85 Gb of useful reads after selection and trimming, respectively. Sequence data were assembled with the Velvet assembler (https://www.ebi.ac.uk/~zerbino/velvet). Gap filling was performed using GapCloser (http://soap.genomics.org.cn/soapdenovo.html) on scaffolds larger than 2 kb. The final assembly resulted in 1 circular scaffold comprising 4 contigs with a cumulative size of 3.84 Mb and a G+C content of 60.8%. Automatic and manual gene annotations were performed using the MicroScope platform (14). The genome contains a single rRNA operon and encodes 47 tRNAs for all amino acids. It features genes for enzymes participating in methanol, methylamine, dimethylamine, and trimethylamine oxidation and associated accessory genes; for enzymes involved in formaldehyde oxidation by the tetrahydromethanopterin pathway, enzymes of the tetrahydrofolate-dependent C1 pathway, and corresponding cofactor biosynthesis genes; and for enzymes involved in formate oxidation, as well as genes of the serine and ethylmalonyl-CoA cycles for carbon assimilation (15). The genome also possesses the strongly conserved *dcm* islet of *dcmRABC* genes found in all bacteria with *dcmA*-encoded DCM dehalogenase (3), including *Hyphomicrobium denitrificans* ATCC 51888 of known genome sequence (16). Genes associated with dissimilatory nitrate reduction and complete denitrification to N_2_ were also identified, as expected from the known ability of *Hyphomicrobium* strains to grow with DCM with nitrate as the terminal electron acceptor in the absence of oxygen (17).

Several other *Hyphomicrobium* genome sequences of isolated strains (16, 18–21) (http://www.genoscope.cns.fr/agc/microscope) or reconstructed from metagenomes (22) are now publicly available. Comparative genomics of the *Hyphomicrobium* genus will support ongoing experimental studies on bacterial adaptation to growth with halogenated methanes for bioremediation applications.

### Accession number(s).

The *Hyphomicrobium* sp. strain GJ21 genome sequence was deposited in GenBank under the accession number CDHO00000000. The version described here is the first version, CDHO01000000.
